# TRPV4 mRNA is elevated in the caudate nucleus with NPH but not in Alzheimer’s disease

**DOI:** 10.3389/fgene.2022.936151

**Published:** 2022-11-02

**Authors:** Hunter White, Ryan Webb, Ian McKnight, Kaitlyn Legg, Chan Lee, Peter H.U. Lee, Olivia Smith Spicer, Joon W. Shim

**Affiliations:** ^1^ Department of Biomedical Engineering, Marshall University, Huntington, WV, United States; ^2^ Department of Anesthesia, Indiana University Health Arnett Hospital, Lafayette, IN, United States; ^3^ Department of Cardiothoracic Surgery, Southcoast Health, Fall River, MA, United States; ^4^ Department of Pathology and Laboratory Medicine, Brown University, Providence, RI, United States; ^5^ National Institute of Mental Health, National Institute of Health, Bethesda, MD, United States

**Keywords:** MRNA marker, TRPV4, MAPT, normal pressure hydrocephalus, Alzheimer’s disease

## Abstract

Symptoms of normal pressure hydrocephalus (NPH) and Alzheimer’s disease (AD) are somewhat similar, and it is common to misdiagnose these two conditions. Although there are fluid markers detectable in humans with NPH and AD, determining which biomarker is optimal in representing genetic characteristics consistent throughout species is poorly understood. Here, we hypothesize that NPH can be differentiated from AD with mRNA biomarkers of unvaried proximity to telomeres. We examined human caudate nucleus tissue samples for the expression of transient receptor potential cation channel subfamily V member 4 (TRPV4) and amyloid precursor protein (APP). Using the genome data viewer, we analyzed the mutability of TRPV4 and other genes in mice, rats, and humans through matching nucleotides of six genes of interest and one house keeping gene with two factors associated with high mutation rate: 1) proximity to telomeres or 2) high adenine and thymine (A + T) content. We found that TRPV4 and microtubule associated protein tau (MAPT) mRNA were elevated in NPH. In AD, mRNA expression of TRPV4 was unaltered unlike APP and other genes. In mice, rats, and humans, the nucleotide size of TRPV4 did not vary, while in other genes, the sizes were inconsistent. Proximity to telomeres in TRPV4 was <50 Mb across species. Our analyses reveal that TRPV4 gene size and mutability are conserved across three species, suggesting that TRPV4 can be a potential link in the pathophysiology of chronic hydrocephalus in aged humans (>65 years) and laboratory rodents at comparable ages.

## Introduction

Normal pressure hydrocephalus (NPH), an abnormal accumulation of cerebrospinal fluid (CSF) in the cerebral ventricles with close to normal intracranial pressure (ICP) (Hill and Volpe, 1981), occurs as the flow of CSF is blocked in the brain and/or spinal cord (Zhang et al., 2019; Han et al., 2021; Tseng et al., 2022). NPH can be found in young adults but is most common in the elderly (Borzage et al., 2021; Buyukgok et al., 2021; Koo et al., 2021; Oike et al., 2021; Mechelli et al., 2022). There are two types of NPH, one (idiopathic NPH, iNPH) with an unknown cause, and another (secondary NPH, sNPH) resulting from a subarachnoid hemorrhage, head trauma, infection, tumor, or complications of surgery (Kiefer and Unterberg, 2012; Williams and Malm, 2016; Gavrilov et al., 2019; Tan et al., 2021; Thavarajasingam et al., 2021; Tseng et al., 2022). A previous survey described the prevalence of NPH as 11/100,000 in adults 19–64 years of age, and 175/100,000 in the elderly over 65 years of age (Isaacs et al., 2018). A more recent review on incidence reported overall crude rates of NPH from 0.07/100,000/year in people aged younger than 60 years to 1.2/1,000 per year in people aged older than 70 years (Zaccaria et al., 2020).

Alzheimer’s disease (AD) is the most common form of dementia and may contribute to roughly 70% of cases (GBD 2019 Dementia Forecasting Collaborators, 2022). In America, as many as 5.8 million individuals had AD in 2020 (Matthews et al., 2019). iNPH is estimated to constitute about 10% of the people diagnosed with a dementia-related disorder, which is expected to exceed 150 million by 2050 (Reeves et al., 2020). NPH and AD share multiple clinical and pathologic features such as amyloid beta (Aβ) deposition, cerebrovascular inflammation, impaired localization of aquaporin 4 (AQP-4), and sleep disturbances (Reeves et al., 2020).

Molecular biomarkers of NPH have been proposed and include amyloid precursor protein (APP) (Miyajima et al., 2013; Pyykko et al., 2014; Jingami et al., 2015; Kazui et al., 2016; Niermeyer et al., 2020; Torretta et al., 2021; Jeppsson et al., 2022) and angiogenic proteins such as vascular endothelial growth factor (VEGF) (Huang et al., 2016) and matrix metalloproteinases (Jeppsson et al., 2022) present in CSF. Markers involved in glymphatic clearance of CSF in the periventricular white matter of the brain have also been proposed (Weiss et al., 2019; Morales et al., 2021; Warsi and Ibrahim, 2021). However, there is no consensus as to whether these markers are specific enough to identify patients with NPH over other age-related conditions such as AD. At present, there is no specific clinically relevant biomarker differentiating NPH (Dewan et al., 2018) from normal aging (Zhang et al., 2021; de Souza et al., 2022; Palmer et al., 2022). Diagnosis remains primarily through neuroimaging with brain scans, which are used for screening, diagnostic, and monitoring purposes (Chatzidakis et al., 2008; Ringstad et al., 2017; Agerskov et al., 2020; Ugga et al., 2020; Virhammar et al., 2022).

The caudate nucleus, a pair of brain structures that reside on the striatal side lateral to the cerebral ventricles, forms part of the basal ganglia and control high-level functions such as planning, movement, and learning in humans (Vergara-Aragon et al., 2011; Karim et al., 2018; Sobue et al., 2018). Because glymphatic system dysfunction evidenced by reduced metabolic clearance appears in NPH and AD alike, the caudate nucleus received much attention as this is the site where the contrast agent is injected (Cserr and Ostrach, 1974) and removal of extracellular marker is monitored (Cserr et al., 1977). In a previous rabbit study by Del Bigio and Bruni, it was demonstrated that experimental hydrocephalus induced by silicone oil injection into the cisterna magna of juvenile rabbits caused a reduction in the number of capillaries in the periventricular region involving the caudate nucleus, while no changes were observed in large blood vessels (Del Bigio and Bruni, 1988). This finding on ‘capillaries’ suggests that vascular endothelia rather than pericytes and smooth muscle cells in the caudate nucleus, primarily contribute to the decreased cerebral blood flow observed in hydrocephalus of neonates and juveniles (Del Bigio and Bruni, 1988; da Silva et al., 1995) and in NPH of aged individuals (Bateman, 2009; Sakellaridis and Stavropoulos, 2010; Sedighi et al., 2014; Ziegelitz et al., 2016; Azuma et al., 2019). Furthermore, caudate structural abnormalities (DeVito et al., 2007), cognitive impairment (Peterson et al., 2019), and striatal dopamine deficit and motor impairment (Pozzi et al., 2021) highlighted the significance of the caudate nucleus in NPH of elderly individuals. The most common form of hydrocephalus in ‘adults’ is iNPH (Williams and Malm, 2016), which is often confused with AD due to similarities in symptoms (Graff-Radford, 2014; Yamada et al., 2016). Although the cognitive impairment can be confused between the NPH and AD, gait dysfunction is a primary differentiating feature, which is present in NPH but not in AD (He D. et al., 2021; He M. et al., 2021; Chunyan et al., 2021; Davis et al., 2021; Hnin et al., 2021; Jeong et al., 2021; Kuruvithadam et al., 2021; Morel et al., 2021; Nikaido et al., 2021; Tang et al., 2021; Yamada et al., 2021; Maier et al., 2022; Nikaido et al., 2022; Sun et al., 2022; Suzuki et al., 2022).

Abnormal tau proteins found in brains cells have been associated with AD and other neurodegenerative diseases (Iqbal et al., 2010). Mutations of genes encoding APP and the p. A152T variant of microtubule associated protein tau (*MAPT*) (Coppola et al., 2012) contribute to the pathogenesis of AD (Mullan et al., 1992) and amyloidogenesis (Di Fede et al., 2009). A673V APP mutations in human brains have shown that amyloid beta deposits are found in all areas of the cerebral cortex, including the caudate nucleus (Giaccone et al., 2010). Although fluid-derived beta-amyloid protein, total tau protein, and phosphorylated tau protein have all been proposed as biomarker proteins of AD (Mulder et al., 2010; Brickman et al., 2022). However, the presence of AD-specific APP but not MAPT (Trabzuni et al., 2012) mRNA, in the caudate nucleus remain elusive (De Meyer et al., 2010).

Transient receptor potential cation channel subfamily V member 4 (TRPV4) is an ion channel protein that is recently being studied as a potential target for congenital hydrocephalus (CH) of neonates. Pharmacological inhibition of TRPV4 in hydrocephalic rats (Shim et al., 2019) ameliorates ventriculomegaly (Hochstetler et al., 2020). In addition to having similar genomic features that are associated with high mutation rates (McKnight et al., 2021), adult mice lacking zinc finger CCHC domain-containing protein 8 (ZCCHC8) (Gable et al., 2019) develop hydrocephalus. Thromboxane A synthase 1 (TBXAS1) is a cytochrome P450 enzyme that catalyzes the conversion of prostaglandin H2 to thromboxane A2, and is a potent vasoconstrictor and inducer of platelet aggregation (Ramazi et al., 2019). TBXAS1 has been reported to improve blood flow and recovery from hindlimb ischemia in mice (Amano et al., 2016), and plays a role in cardiovascular disease, stroke, and dementia (Ramazi et al., 2019; Bhatia and Singh, 2021a; b). However, whether these molecules at the mRNA level can serve as biomarkers of NPH and/or AD remains unsubstantiated.

While a larger scale analysis of the whole transcriptome undoubtedly requires higher (at least six) replicates, we have embarked on an idea that downsizing into select genes would allow immediate screens of the unknown such as ZCCHC8 as compared to a widely investigated gene like APP, given the repetitive failures of clinical trials for therapies of AD (Anderson et al., 2017).

The goal of this study was to quantify relative mRNA levels of TRPV4, ZCCHC8, VEGF, TBXAS1, MAPT, and APP, in postmortem human caudate nucleus samples from elderly (>66 years of age) cadaveric donors previously diagnosed with NPH and AD as compared to unaffected controls. We also examined the mutable characteristics of the various genes across species, comparing human, mouse, and rat genomes. Finally, we investigated across species whether mRNA expressions of these genes correlate with their relative mutability through two factors associated with high mutation rates.

## Results

**FIGURE 1:**
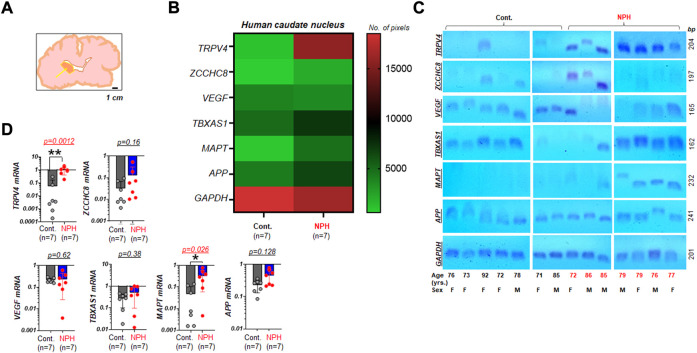
Elevated TRPV4 mRNA in the caudate nucleus with NPH **(A)** A simplified illustration indicating the neuroanatomical location where human postmortem tissue was harvested for assays (arrow: the caudate nucleus). The illustration was redrawn following the photograph taken at one of NeuroBioBank (NBB) tissue repositories. Scale bar, 1 cm **(B)** a color-coded map illustrating the summary of the assay result on expression levels of TRPV4 and other genes associated with NPH in the caudate nucleus of humans with unaffected control (Cont.) and NPH, respectively. Note that 6 genes of interest and 1 housekeeping gene (GAPDH) were marked on the left *y* axis. The number of pixels from ImageJ with coded color was shown on the right. *n* = 7 per group. **(C)** Agarose gels displaying mRNA expressions of TRPV4, ZCCHC8, VEGF, TBXAS1, MAPT, and APP relative to GAPDH in the caudate nucleus of aged human postmortem brains classified as Cont. (n = 7) and NPH (n = 7). yrs., years **(D)** bar graphs with scatter plots summarizing mRNA expressions of select genes relative to GAPDH in NPH (n = 7) as compared to those of unaffected controls (n = 7). **, *p* < 0.01; *, *p* < 0.05 by Mann-Whitney test.

We investigated the mRNA expression of six genes relative to the internal reference, GAPDH, in caudate nucleus samples harvested from postmortem brains of aged humans with NPH. These tissues had been preserved as frozen samples and were obtained from multiple repositories of the NIH NBB (Figure 1A). Expression patterns of the six genes from the set of samples that arrived from the biobank were assessed using RT-PCR (n = 7 per group). All six genes of interest appeared to demonstrate differential mRNA expressions in NPH compared to control samples (Figure 1B). In a subsequent image analysis on DNA agarose gels (Figure 1C), we found that mRNA expressions of TRPV4 and MAPT were significantly elevated in the caudate nucleus samples of NPH (n = 7) as compared to those of unaffected controls (n = 7) (Figures 1C,D).

**FIGURE 2:**
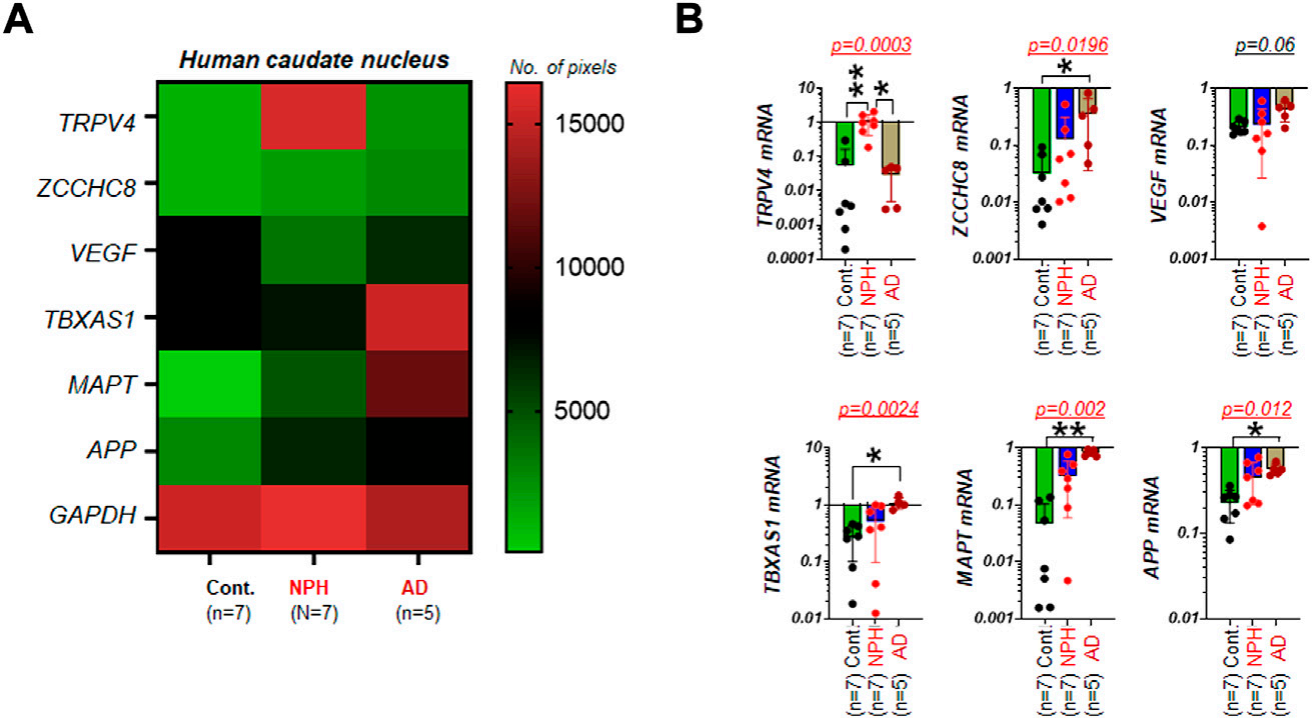
TRPV4 mRNA in the caudate nucleus is elevated in NPH but not in AD **(A)** A color-coded map illustrating the summary of the mRNA assay result on expression levels of TRPV4 and five other genes associated with either NPH or AD in the caudate nucleus of humans with unaffected control (Cont.), NPH, and AD, respectively. The number of pixels from ImageJ with coded color was shown on the right. Note that TRPV4 and VEGF mRNA were not altered in AD as compared controls. N = 5-7 per group. **(B)** bar graphs with scatter plots summarizing mRNA expressions of multiple genes relative to GAPDH in AD (n = 5) as compared to those of Cont. (n = 7). **, *p* < 0.01; *, *p* < 0.05 by Kruskal-Wallis test.

We next tested whether those genes assayed for NPH (Figure 1) are differentially regulated in the caudate nucleus of aged humans with AD. In the mRNA assays by RT-PCR with the set of samples obtained from the biobank, several genes of interest appeared to demonstrate altered gene expressions in NPH and/or AD as compared to unaffected controls (Figure 2A). In the image analyses of DNA agarose gels (Supplementary Figure S1), we found that mRNA expressions of ZCCHC8, TBXAS1, MAPT, and APP in AD (n = 5) were significantly elevated as compared to those of controls (n = 7). Unlike these four genes, mRNA level of VEGF was not significantly altered as compared to that of controls. TRPV4 mRNA in the caudate nucleus was elevated in patients with NPH but not in the cases with AD as compared to unaffected controls (Figure 2B).

**FIGURE 3:**
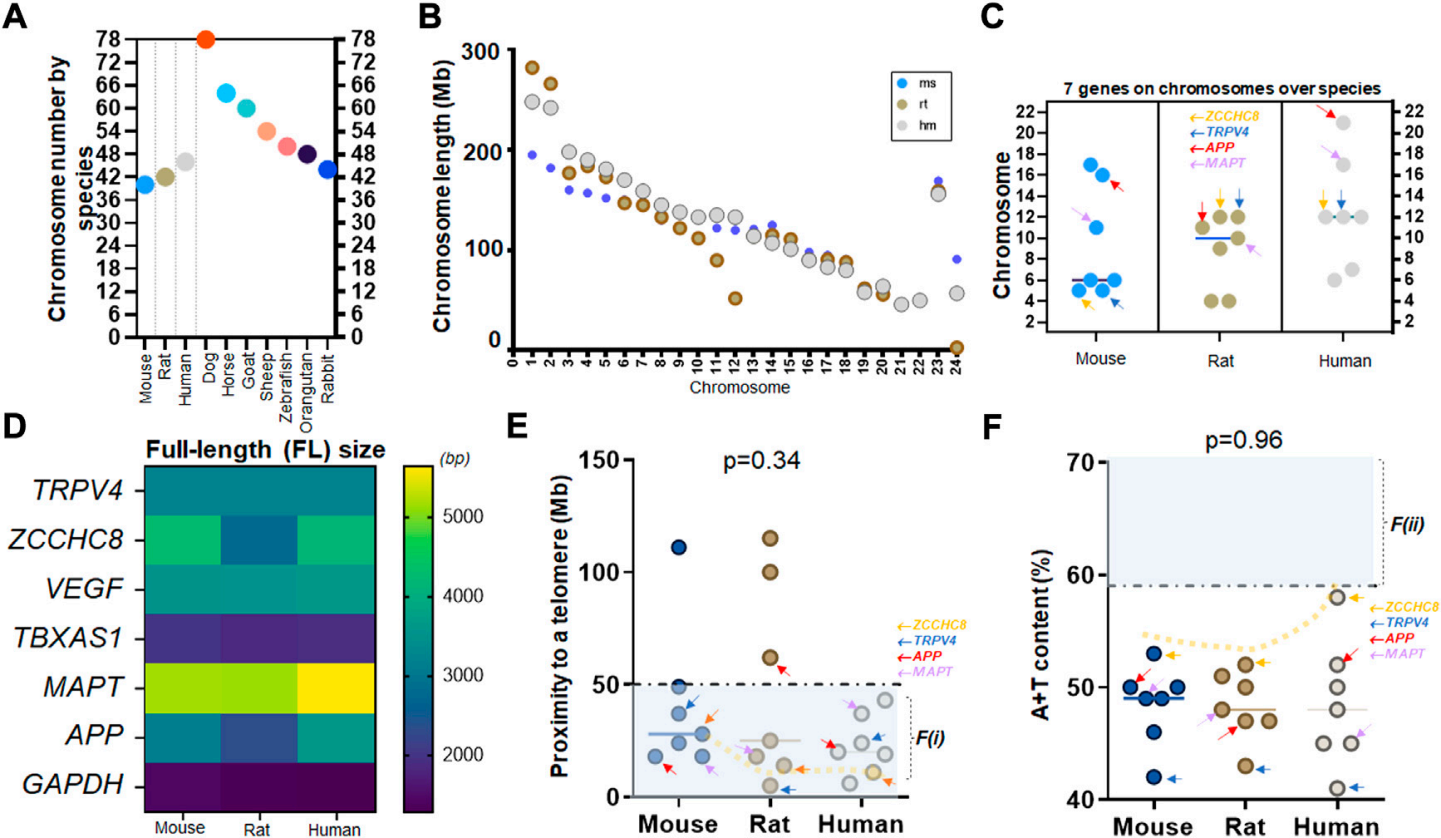
A consistent mutability of TRPV4 over mouse, rat, and human genomes **(A)** Chromosome numbers of frequently used laboratory animals (mice and rats) and humans as compared to other species such as dogs to rabbits. Note that orangutan (48) and rabbit (44) have similar numbers to humans at 46 chromosomes (23 pairs) while dogs are much higher at 78. **(B)** Scatter plots showing chromosome length of three species with respect to each chromosome (chr) of chr 1 to X or Y; mice (ms), rats (rt), and humans (hm). Note that larger animals do not always show longer chromosomes. In chr 1, rats, which are smaller in body size, have longer nucleotide sizes than humans. *x*-axis is pair-wise; humans showing 23 pairs from chr 1 to chr X or Y (Morton, 1991) **(C)** Locations of seven genes investigated in this study over three species of mouse, rat, and human. Note that APP and MAPT have evolved into shorter chromosomes (chr17-22) in humans as compared to rodents **(D)** The full-length (FL) size of seven genes over three species. Note that TRPV4, VEGF, and TBXAS1 show consistent molecular sizes over three species while ZCCHC8, APP, and MAPT differ in humans as compared to rodents. **(E)** Proximity to telomeres of seven genes over three species. Note that seven human genes investigated in this study have evolved in a way meeting proximity to telomeres or the first factor, F(i), associated with high mutation rate as all seven human genes are at less than 50 Mb as compared to those of mice and rats. **(F)** A+T content of seven genes over three species. Seven human genes demonstrate a similar characteristic of difficulty in meeting this second factor, F(ii), associated with high mutation rate while ZCCHC8 gene has evolved towards 59%. A dotted trend line follows ZCCHC8 gene in mice, rats, and humans **(E,F)**. Arrows in orange, blue, red, and purple indicating ZCCHC8, TRPV4, APP, and MAPT, respectively **(C,E,F)**.

We have previously shown that there are two factors based on gene characteristics that are associated with high mutation rates (Nusbaum et al., 2006), and can be used to assess the relative mutability of genes associated with various human diseases (Lucas et al., 2021; McKnight et al., 2021) and those targeted by specific drugs (Raines et al., 2022). These two factors are proximity to telomeres and high A + T content and were proposed in studies of human chromosomes (Nusbaum et al., 2006). Using these two factors as a screening tool, we asked if the mutability of the six genes of interest with one housekeeping gene we assayed from the caudate nucleus of human samples are similar or different across mammalian species. Using NCBI genome resources (txid9606 [orgn]), we organized the chromosome count of humans and other mammalian species such as mouse, rat, dog, horse, goat, sheep, zebrafish, orangutan, and rabbit, which are frequently used in the laboratory investigation. Due to differences in chromosomal numbers, the organized data suggest that the chromosomal location of specific genes could vary widely across species. For instance, orangutans and rabbits have similar chromosome counts as humans at 46 (23 pairs) while dogs have roughly twice as many chromosomes (Figure 3A).

To gain a better understanding of the association of telomere proximity with physical characteristics in various species, we first plotted the nucleotide or transcript length of chromosomes between mice, rats, and humans, and showed that body size is not necessarily linearly associated with chromosome size. Evidently, chromosome #1 (chr 1) is the longest among all chromosomes in these three species but the chromosome length by species does not conform to the rank order of the body size. In other words, even though humans are significantly bigger than rats, the length of chr 1 in rats (∼300 Mb) is longer than that of humans (∼250 Mb) (Figure 3B). We then compared the distributions of the six genes of interest and one housekeeping gene in three species: mice, rats, and humans (Supplementary Tables S1.1–S1.3). Given that the three species share similar counts of chromosome pairs at 20 (mouse), 21 (rat), and 23 (human), we found that genes were on different chromosomes depending on species. Taking the observed peak value (chr 21 for APP, marked in red arrows) in humans for example, the same gene is at chr 16 in mice, while APP gene in rats is at chr 11 (arrows in red; Figure 3C). Two known fluid biomarker genes of AD, APP and MAPT, are found on much shorter chromosomes in humans compared to mice and rats. (Figures 3B,C). Contrastingly, the location of ZCCHC8 and TRPV4 genes, did not jump to chr 20 or higher (relatively shorter chromosomes) in the human genome, suggesting no abrupt relocation from the longer sized chromosomes (chr 1–8) to the intermediate ones (chr 9–16) in mouse and rat genome (Figure 3C).

We next assessed the nucleotide (transcript) size of the seven genes in mice, rats, and humans. By color-coding, we found that TRPV4, VEGF, TBXAS1, and GAPDH nucleotide size are consistent over the three species (Figure 3D). Two known fluid markers of AD, MAPT and APP demonstrated a longer nucleotide size in the human genome as compared to mice and rats (Figure 3D). Assessments of proximity to telomeres suggest that while most genes across species satisfied proximity to telomeres at <50 Mb, it was only in humans where all seven genes met this criterion (Figure 3E). Although one nucleotide difference in mouse and human isoform such as VEGF-A_164_ in mice pertaining to VEGF-A_165_ in humans is well appreciated, elongated nucleotide sizes in APP and MAPT of humans compared to mice and rats were suggestive of higher GC content in APP and MAPT gene. Indeed, APP gene showed the highest A + T content in human genome as compared to mouse and rat genome (Figure 3F).

**FIGURE 4:**
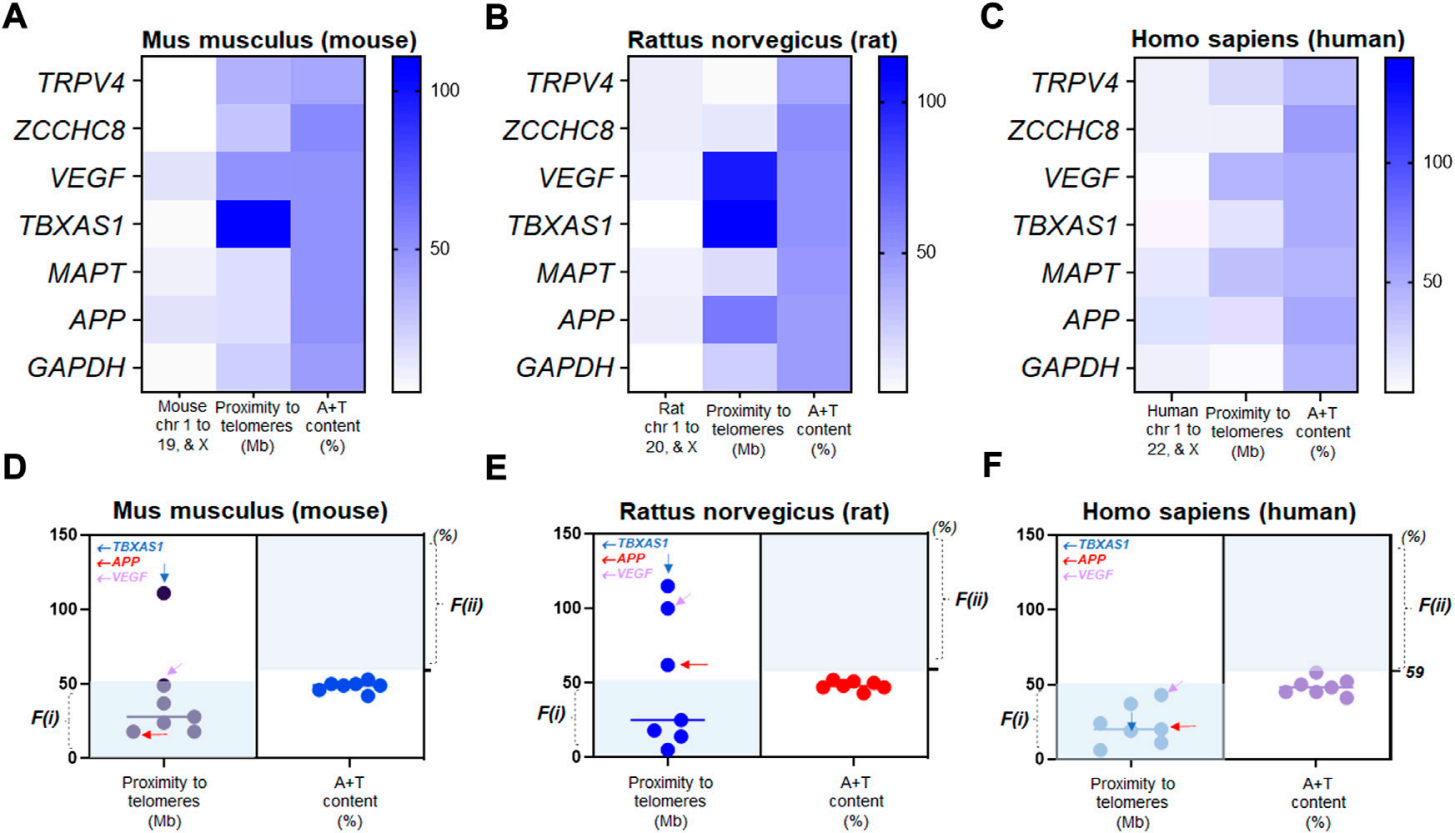
Relative mutability in human genes driven by proximity to telomeres **(A)** The color-coded map showing chromosomal location, distance to a telomere, and A+T content of *Mus musculus* (mouse) TRPV4 and other genes in chromosome numbers (chr 1 to 19 and X), proximity to telomeres (Mb), and A+T content (%). **(B)** the color-coded map showing chromosomal location, distance to a telomere, and A+T content of *Rattus norvegicus* (rat) TRPV4 and other genes in chromosome numbers (chr 1 to 20 and X), proximity to telomeres (Mb), and A+T content (%). **(C)** the color-coded map showing chromosomal location, distance to a telomere, and A+T content of *Homo sapiens* (human) TRPV4 and other genes in chromosome numbers (chr 1 to 22 and X), proximity to telomeres (Mb), and A+T content (%). **(D)** Scatter plots displaying relative mutability of seven mouse genes by proximity to telomeres (left) and A+T content (right). Shaded regions with light colors (blue) indicate genes satisfying either of the two factors. Genes outside the shaded regions at n = 2 by F(i); n = 7 by F(ii)) are relatively less mutable. **(E)** Scatter plots displaying relative mutability of seven rat genes by proximity to telomeres (left) and A+T content (right). Shaded regions with light colors (blue) indicate genes satisfying either of the two factors. Genes outside the shaded regions at n = 3 by F(i); n = 7 by F(ii)) are relatively less mutable. **(F)** Scatter plots displaying relative mutability of the same seven genes by proximity to telomeres (left) and A+T content (right) in human chromosomes. Shaded regions with light colors (blue) indicate human genes satisfying either of the two factors. Genes outside the shaded regions at n = 0 by F(i); n = 7 by F(ii)) are relatively less mutable. Note that all human genes satisfied proximity to telomeres, F(i), while none met F(ii).

Next, we examined the alterations of two factors in seven genes we assayed for NPH and AD over three species. Using color-coded plots, we organized these genes relative to chromosomal location, proximity to telomeres, and A + T content in mouse, rat, and human genome. We found that chromosomal characteristics of VEGF, TBXAS1, and MAPT differ depending on species (Figures 4A–C). As to the genetic mutability identified by proximity to telomeres, mouse TBXAS1 gene was relatively less mutable as this gene failed to satisfy F(i) (Figure 4D). In the rat genome, as we assessed the mutability of seven genes by proximity to telomeres, TBXAS1, APP, and VEGF were relatively less mutable as these three genes did not meet F(i) (Figure 4E). In human chromosomes, seven genes were relatively much more mutable by proximity to telomeres as these genes satisfied F(i). The mutability of seven genes demonstrated a consistent trend by A + T content as all genes failed to satisfy F(ii) over species (Figures 4D–F). In humans, TBXAS1, APP, and VEGF were located in more proximity to their telomeres at <50 Mb as compared to rodent genomes, suggesting that proximity to telomeres of these genes are not consistent when it comes to human chromosomes as compared to those of mice and rats (Figure 4F).

To further investigate TRPV4 localization in human cells, we cultured vascular EC line and culture over 14 days (Supplementary Figure S2). When TRPV4 was co-labeled with α tubulin, a marker for microtubules, areas of the TRPV4 positive immunofluorescence were conversely associated with areas of the α tubulin positive immunoreactivity in these cells *in vitro* (Supplementary Figures S2A,B). This finding was consistent with an inverse relationship between the microtubule length in radially spanning elliptic formation of α tubulin in the cytoplasm and DAPI-positive cell numbers over 14 days of the culture (Supplementary Figures S2C,D). This finding on microtubule elongation/shortening in TRPV4-positive cellular phenotype was consistent with our observation of ependymal cilia in neonatal rat brains *in vivo* (Shim et al., 2019). In human vascular ECs, there were two distinct patterns of TRPV4 proteins near cytoplasm and cell nucleus. Specifically, the area of TRPV4-positive immunoreactivity was significantly decreased (*p* = 0.002) in human ECs with nuclear TRPV4 as compared to those with cytoplasmic TRPV4 (Supplementary Figures S2E–J).

## Discussion

We demonstrated from mRNA assays of the caudate nucleus specimens obtained from aged cadavers that TRPV4 has the potential to be a novel tissue marker of NPH, although follow-up studies with a larger same size are warranted (Figure 1). Moreover, TRPV4 expression is specifically elevated in NPH but not in AD. Our finding of elevated expression of APP in AD caudate nucleus is consistent with the expected elevations of these markers in the plasma or CSF of individuals with AD (Brickman et al., 2022). The finding that MAPT mRNA is elevated in the caudate nucleus from NPH as well as AD patients suggests that NPH and AD are not differentiated with MAPT alone, if used as an mRNA biomarker. Additionally, the elevated *TBXAS1* mRNA expression levels we found in these AD tissues bolsters the finding of the previous report (Grubman et al., 2019) (Figure 2).

While the plethora of recent articles can be retrieved as one uses the search keyword of ‘biomarker and hydrocephalus’ (Nakajima et al., 2010; Miyajima et al., 2013; Pyykko et al., 2014; Jingami et al., 2015; Kazui et al., 2016; Nagata et al., 2017; Abu-Rumeileh et al., 2019; Niermeyer et al., 2020; Nakajima et al., 2021; Torretta et al., 2021; Daly et al., 2022; Jeppsson et al., 2022; Mazzeo et al., 2022), we have not yet found literature focusing on ‘mRNA biomarker and hydrocephalus’. Instead, there were two recent articles related to ‘mRNA biomarker and Alzheimer’s (Fiala et al., 2011; Isik and Beydemir, 2022), suggesting that most biomarker studies on iNPH and/or sNPH might be designed through fluid or CSF assays (Nakajima et al., 2010; Miyajima et al., 2013; Pyykko et al., 2014; Jingami et al., 2015; Kazui et al., 2016; Nagata et al., 2017; Abu-Rumeileh et al., 2019; Niermeyer et al., 2020; Nakajima et al., 2021; Torretta et al., 2021; Daly et al., 2022; Jeppsson et al., 2022; Mazzeo et al., 2022). This highlights the novelty and utility of tissue assays in which mRNA markers were pursued with the caudate nucleus.

In our unbiased assortation of chromosome numbers in ten species, we found, as expected, that primates (orangutans) have chromosome numbers consistent with those of humans. However, dogs, a species commonly used for neurosurgical studies (Dombrowski et al., 2006; Park et al., 2010; Gomes et al., 2020b; a;Hasegawa, 2020; Olszewska et al., 2020; Ito et al., 2021; Lee et al., 2021), have significantly more chromosomes (78), suggesting reconsiderations regarding the appropriateness of the canine model for certain genetic studies.

We also report that one of the highly used housekeeping genes, GAPDH, maintains a consistent nucleotide size across mouse, rat, and human genomes, along with TRPV4, VEGF, and TBXAS1 genes (Figure 3). In evaluating the mutability factor F(i) (Lucas et al., 2021; McKnight et al., 2021; Raines et al., 2022) in the three species assessed, we did not expect biomarker signatures or mutability of TBXAS1, APP, and VEGF genes to be similar in mice and rats as compared to humans. However, contrarily, mutability of MAPT, ZCCHC8, and TRPV4 genes were found to be consistent across mice, rats, and humans (Figures 4E,F) except molecular sizes (Figure 4D).

The findings of clearly elevated MAPT and APP mRNA in AD samples compared to controls suggest the specificity of tau protein and amyloid beta as potential caudate nucleus markers of AD. Although the antibody-based detection method is still available, the diagnostic utility of PCR has risen since the COVID-19 pandemic. Our mRNA assay with the caudate nucleus suggests t*hat MAPT,* when combined with multiple genes such as APP and TBXAS1, is a more specific way of distinguishing AD compared to using only a single or dual markers. Our data, based on human caudate nucleus tissue samples from postmortem brains provide useful insight into novel potential tissue markers of AD, Firstly, TBXAS1 mRNA, a marker of platelet aggregation or atheroprone vasculature (Ramazi et al., 2019) is positively detected in all caudate nucleus samples of AD, while TBXAS1 mRNA is not always detectable in controls. Secondly, mRNA of VEGF-A_165_, which is indicative of dysfunctional vasculatures or insufficient angiogenesis (Shim and Madsen, 2018) required for the maintenance of adult brains (Echeverria et al., 2017), did not alter in the caudate nucleus from NPH and AD alike. When unaffected control tissue (the caudate nucleus) is not available but specimens from patients such as those with AD and NPH are available, MAPT mRNA in combination with APP, TBXAS1, and ZCCHC8 can distinguish AD with higher accuracy than with a single biomarker.

Here, the utility of the caudate nucleus as a target specimen for mRNA marker detection is demonstrated but the assay should be extended to other regions of the brain to overcome the inherent limitation. In AD, there are prior studies measuring mRNA or microRNA in the blood (Kester et al., 2011; Lu et al., 2018) or CSF (Gui et al., 2015). We confirmed the previous finding that MAPT and APP mRNA are elevated in the caudate nucleus (tissue), consistent with fluid assays of AD (Musiek and Holtzman, 2012; Lafirdeen et al., 2019; McLimans et al., 2019; Barthelemy et al., 2020; Shen et al., 2020; Shen et al., 2021). With the sample size applied in the current findings, it is not yet reasonable to predict or utilize other mRNA markers as a diagnostic tool for AD (compared to control) given the number of other studies that have examined far larger cohort sizes (Georganopoulou et al., 2005; Holtta et al., 2013). Although assaying mRNA in the blood (Ekici et al., 2010) or CSF with NPH has been rarely reported, we found that an attempt has been made to detect microRNA in the CSF of patients with congenital hydrocephalus (Chen et al., 2022). Nevertheless, we envision that the data presented in this report on a more specific marker gene like TRPV4 and MAPT in NPH can significantly improve the prior cases reporting misdiagnosis of either AD or NPH (Joseph et al., 2006; Joseph et al., 2008; Lee et al., 2010; Shimada et al., 2011). Biomechanical aspects of intracranial compliance (Gholampour et al., 2022) should also be considered in the future as well for NPH and AD biomarker studies.

There are immunological cells such as T lymphocytes in the CSF (Evans et al., 2019) that can be analyzed by single cell techniques (Esaulova et al., 2020; Guan et al., 2022; Piper et al., 2022; You et al., 2022). To study CSF and compare that with the brain tissue finding is thus highly relevant. This is important since one cannot yet expect deep brain biopsy (Yu et al., 1998; Yamada et al., 2004; Moriuchi et al., 2011; Ritz et al., 2011; Ooba et al., 2013; Iijima et al., 2015; Wang et al., 2016; Arakawa et al., 2020; Qin et al., 2021; Tang and Wang, 2021; Zhu et al., 2021) to be a realistic diagnostic tool to distinct either iNPH or sNPH from AD. Furthermore, there are already less invasive biomarkers, from brain imaging and CSF, available for that (Nakajima et al., 2010; Torretta et al., 2021; Jeppsson et al., 2022; Mazzeo et al., 2022). To suggest TRPV4 as a biomarker for differential diagnostics, it is thought that there should be a larger replication cohort.

In conclusion, TRPV4 mRNA in the caudate nucleus is elevated in NPH but not in AD. The result suggests that TRPV4 can be a potential link in the pathophysiology of chronic hydrocephalus in aged humans (>65 years) and laboratory rodents at comparable ages.

## Methods

### Human postmortem tissues

Postmortem tissues were requested from the National Institute of Health (NIH) NeuroBioBank (NBB), USA over a period of 1 year. We collected the postmortem tissues of aged individuals through multiple repositories of the NIH NBB, which provided the caudate nucleus (DeVito et al., 2007; Deshpande et al., 2009; Jang et al., 2017; Peterson et al., 2019) (Figure 1A) in a frozen state. Caudate nucleus specimens in frozen state were transported to our lab. Per the record provided by the NBB, the specimens were collected at postmortem intervals of 16 ± 8 h (mean ± std; range 4–25 h after death, n = 7 in unaffected controls; n = 7 in NPH; n = 5 in AD). Inclusion criteria and diagnosis are provided in Supplementary Tables S2, S3. Seven male and twelve female brain specimens are used, where sex is noted in Supplementary Figure S1 and Supplementary Table S3.

### Primer design

We designed the primers for six genes of interest with one housekeeping gene based on the prior reports (Giaccone et al., 2010; Trabzuni et al., 2012; Mesitskaya et al., 2018; Shim and Madsen, 2018; Gable et al., 2019; Cacabelos, 2020; Hochstetler et al., 2020). Human gene transcripts were searched using Ensembl database (http://useast.ensembl.org/index.html). Using Primer3 online, we determined the sequences of a specific exon per gene transcript (https://bioinfo.ut.ee/primer3-0.4.0/). Then, lyophilized forms were manufactured and provided by the vendor (Thermofisher scientific, Waltham, MA). Seven human gene primers were designed (Supplementary Table S4).

### Total RNA isolation and cDNA generation

Total RNA was isolated from the caudate nucleus of the unaffected control, NPH, and AD specimens using QIA-ZOL based RNA isolation kit (RNeasy Lipid Tissue Mini Kit, QIAGEN). Concentration and quality of samples were analyzed with a NanoDrop spectrophotometer (Thermofisher). Total RNA (500 ng/reaction) was reverse-transcribed using the High-Capacity RNA-to-cDNA Kit (Thermofisher; Catalog number: 4368814) at ABI SimpliAmp Thermal Cycler System (Thermofisher).

### Reverse transcription polymerase chain reaction

RT-PCR was carried out in 25 μL containing 250 ng cDNA following the manufacturer’s instructions (GoTaq®Green Master Mix). Cycling conditions were composed of three steps: Denaturation at 95 °C for 2  min, followed by 32 cycles of denaturation at 95°C for 30 s, annealing at 60 °C for 30 s, and extension at 72 °C for 30 s (Promega, Madison, WI). The PCR products were then separated by electrophoresis on horizontal 1.25% agarose gels in 1x Tris/boric acid/EDTA (TBE) buffer and visualized by staining with Maestro dye (MaestroSafe, Maestrogen). The fluorescent signal was photographed with the built-in camera of an iPhone 12 (Apple).

### ImageJ analysis

The image analysis of the DNA agarose gels was conducted using NIH ImageJ. The process is composed of six steps: 1) Open gel image at ImageJ, 2) Select with rectangle, 3) Analyze-gels-1st lane and the next until the end, 4) Analyze-gels-plot lanes, 5) Connect with straight lines, and 6) Select with points. The area calculated per each band was collected in the result file and saved in the spreadsheet. The relative fold change for each gene of interest was quantified with respect to the housekeeping gene (Schneider et al., 2012; Shim et al., 2013; Shim et al., 2016).

### Statistical analysis

Statistical analyses were performed using Prism (version 9.3.0, GraphPad Software Inc.), enabling the generation of a heatmap plot and bar graphs of the data obtained during analysis with ImageJ. The non-parametric test was employed as this approach can reach a more conservative conclusion than a parametric test. As a result, a Mann-Whitney test and Kruskal-Wallis test were used for two group and three group comparisons, respectively. The difference between data sets was considered significant at *p* < 0.05; *p* values are identified in the figures and legends as *p < 0.05, **p < 0.01, and ***p < 0.005.

## Data Availability

The original contributions presented in the study are included in the article/Supplementary Material, further inquiries can be directed to the corresponding author/s.
