# Supplementary material for: TRPV4 mRNA is elevated in the caudate nucleus with NPH but not in Alzheimer’s disease
Source: Front Genet. 2022 Nov 2;13:936151. doi: 10.3389/fgene.2022.936151 (PMC9670164; doi:10.3389/fgene.2022.936151)
Supplement: Supplementary file 1 [file DataSheet1.pdf]

Supplementary Material for

TRPV4 mRNA is elevated in the caudate nucleus with NPH but not in Alzheimer's disease

Hunter White[1], Ryan Webb[1], Ian McKnight[1], Kaitlyn Legg[1], Chan Lee[2], Peter H.U. Lee[3,4], Olivia Smith Spicer[5], Joon W. Shim[1*]

Correspondence to: shim@marshall.edu

This file includes:

Supplementary methods,
Figure S1 to S2,
Tables S1 to S4

Supplementary methods:

Cell culture

Human endothelial cells were purchased (EA.hy026, ATCC) and acquired in a frozen state. The frozen cell was thawed at 37°C for two minutes. Then, cell suspension (1 ml) in a cryogenic vial was resuspended with 9 ml of the fresh low glucose Dulbecco's Modified Eagle's Medium (DMEM: Gibco; Carlsbad, CA) containing 10% fetal bovine serum (FBS; BD Biosciences; San Jose, CA) and 1% 1× antibiotic-antimycotic (ABAM) preheated at 37°C. Cells in fresh medium were then placed in Falcon ® flask (Thermo Scientific, Waltham MA) and roughly 50% of the cells were attached to the plate in 2 hrs. The next day, >90% of previously suspended cells were attached to the bottom of the flask and grew without medium change for a week. Next, 5 x 10^5 cells/ml counted at hemacytometer were split into the 6-well plate pre-incubated with a coverslip at the bottom of each well marked as day 1 of the experimental period. From this point forward, cells were cultured with fresh new medium for two weeks and cells on each coverslip was harvested on day 3, 7, 10, and 14 for immunocytochemistry.

Immunofluorescence

Cells (EA.hy026, ATCC) grown on the coverslip in the 6-well plate were transferred to the new well of the empty 6-well plate. The coverslips on which cells grow were then washed with phosphate-buffered saline (PBS) and fixed with 4% paraformaldehyde (PFA) in PBS at room temperature for 2 hrs. Cells were then washed briefly with PBS and treated with blocking solution containing 10 % goat serum and 0.05% Triton-X 100 in PBS at 4°C for a day. Next, the blocking solution was aspirated out and cells were treated with the primary antibody (anti-Trpv4, 1:500; alomone labs #ACC-034 or anti-CD31, 1:500; abcam #ab28364) dissolved in fresh blocking solution covered with the light protectant (foil) overnight at 4°C. Cells were then washed with PBS containing 0.1% tween 20 (PBS-T20) twice (10 min/each wash) and PBS (the third wash). Next, cells were treated with the secondary antibody (goat anti-rabbit cy3; 1:1000; Invitrogen) dissolved in blocking solution at room temperature for 1 hr. The same procedure is taken for the second primary antibody (anti-α tubulin, 1:1000; SigmaAldrich #TS168)

and washed with PBS-T20 and then PBS. Then, cells on the coverslip were transferred to the glass slide with the tweezer and mounted with the mounting media containing 4',6-diamidino-2-phenylindole (DAPI) visualizing nuclear DNA. Mounted cells on the slides were dried overnight within the staining box and imaged under the fluorescence Keyence (BZ-X800) microscope with DAPI (wavelength at 405 nm), Alexa488 (488 nm), and cy3 (532 nm) filter.

Distance to a telomere and nucleotide composition calculation

To obtain the distance from the gene of interest to its telomere, and to calculate A+T content percentage of nucleotides, we utilized the NCBI Genome Data Viewer (https://www.ncbi.nlm.nih.gov/genome/gdv/) and the GC content calculator (https://www.biologicscorp.com/tools/GCContent/#.XvctCi-z2uV). This allows to acquire compositions of adenine and thymine as well as the full-length base-pair sizes of the nucleotide (Lucas et al., 2021;McKnight et al., 2021;Raines et al., 2022).

Approximation of the first factor or the proximity to telomeres

Previously, Nusbaum and colleagues (Nusbaum et al., 2006) have described the biological basis for the high mutation rate in human chromosomes. In this study, we have adopted this established method based on their suggestion (Nusbaum et al., 2006) when we approximate the proximity to a telomere in a gene (Lucas et al., 2021;McKnight et al., 2021;Raines et al., 2022). In the present study, we have calculated the A+T content of the seven genes in mouse, rat, and human chromosome and identified the location of the gene within each chromosome and the locus of the corresponding telomere with the premise below:

If recombination frequency is shorter than (≤) 50 centimorgan (cM), genes are linked; (1)

if recombination frequency is longer than 50 cM, genes are not linked; (2)

where 1 cM ≅ 1 million base pairs (Mb) (Hastbacka et al., 1992)

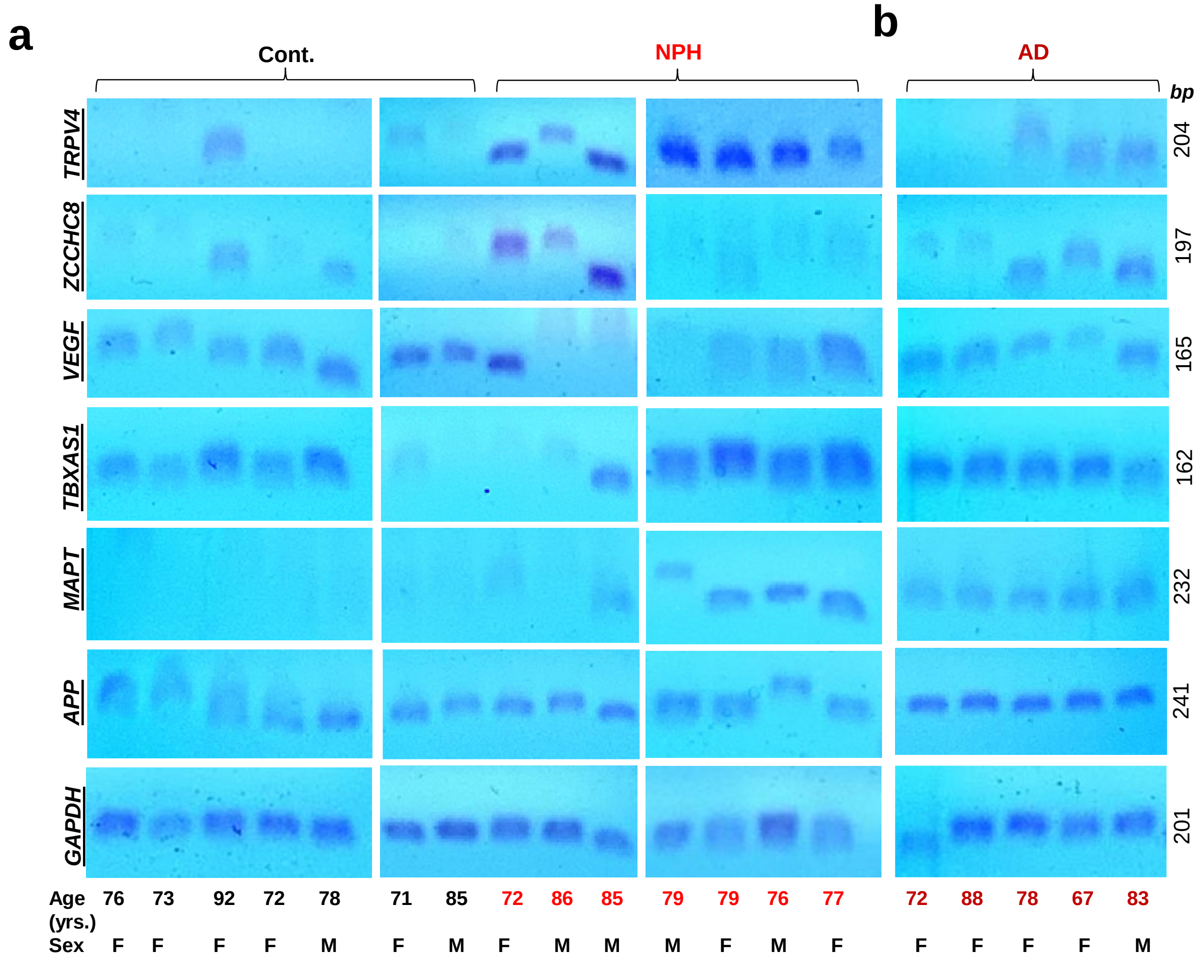

Figure S1. Relative mRNA levels of five marker genes in the caudate nucleus. (a) Agarose gels displaying mRNA expressions of TRPV4, ZCCHC8, VEGF, TBXAS1, MAPT, and APP relative to GAPDH in the caudate nucleus of aged human postmortem brains classified as Cont. (n=7) and NPH (n=7) as shown in Fig. 1. (b) Agarose gels displaying mRNA expressions of TRPV4, ZCCHC8, VEGF, TBXAS1, MAPT, and APP relative to GAPDH in the caudate nucleus of aged human postmortem brains with AD (n=5) as compared to Cont. (n=7); yrs., years; F, female; M, male

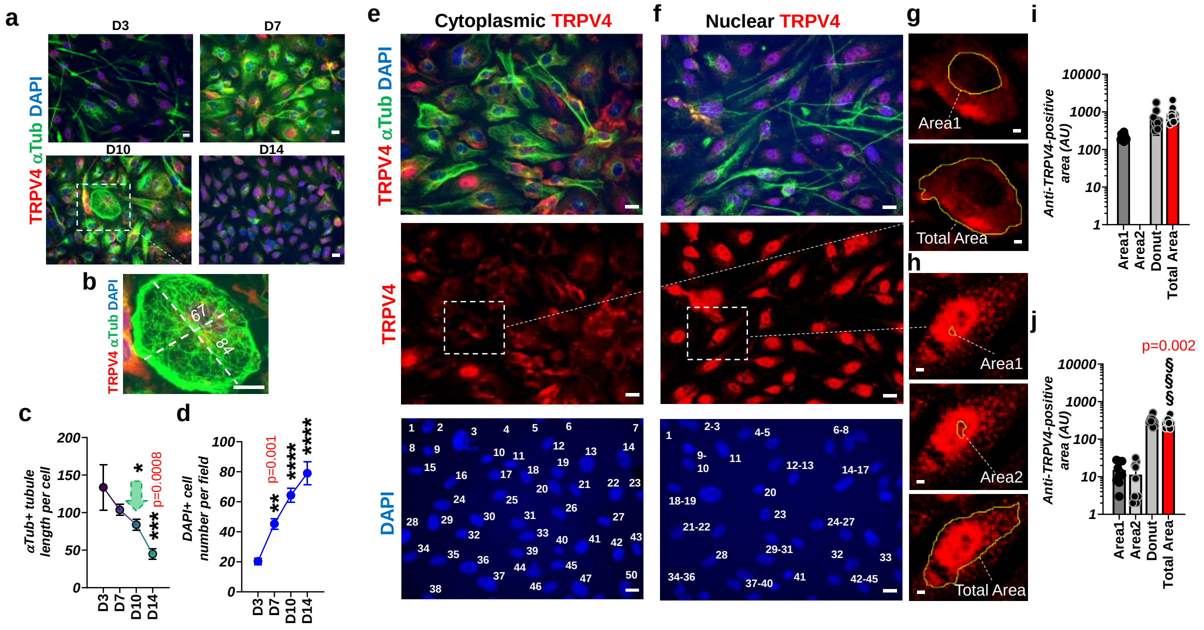

Figure S2. Identification of TRPV4 localization in human vascular ECs (a) Representative micrographs showing anti-TRPV4 and anti-αtubulin immunoreactivity on culture day 3 (D3), day 7 (D7), day 10 (D10: rectangular inset magnified in b), and day 14 (D14) (b) Short (67) and long (84) axis as measured in the elliptic localization of αtubulin in the human vascular ECs on culture D10 (Numbers in arbitrary unit). (c) Average data summarizing span-wise length of microtubules by anti-αtubulin immunofluorescence shown in a on D3, D7, D10, and D14. *, ***; P<0.05, P<0.005 by Mann-Whitney test as compared to the data on D3. Arrow indicates the average value shown in a. (d) Average data summarizing DAPI-positive cell nuclei per field shown in a on D3, D7, D10, and D14. **, ****; P<0.01, P<0.0001 by Mann-Whitney test as compared to the data on D3. (e) Micrographs showing overlay of anti-TRPV4, anti-αtubulin, and DAPI fluorescence (top), anti-TRPV4 by Cy-3 (middle: rectangular inset magnified in g), and DAPI (bottom: numbers indicating cell count) on d10, demonstrating cytoplasmic localization of TRPV4. (f) Micrographs showing overlay of anti-TRPV4, anti-αtubulin, and DAPI immunofluorescence (top), anti-TRPV4 alone (middle: rectangular inset magnified in h), and DAPI (bottom: numbers indicating cell count) on d10, demonstrating localization of TRPV4 in the cell nuclei and peri-nucleus membrane. (g) Cy-3 positive immunofluorescence of TRPV4 in human vascular ECs quantified by subtracting TRPV4-negative puncta (Top: Area 1) from the total area (bottom), resulting in a donut like localization of proteins. (h) Cy-3 positive immunofluorescence of TRPV4 in human vECs quantified by subtracting smaller puncta (Top: Area 1) and larger puncta (Middle: Area 2) out of the total area (bottom), resulting in a donut shape with two inner holes. Scale bars, 20 μm (a-b; e-j). (i) bar graphs with scatter plots summarizing areas of anti-TRPV4-positive immunofluorescence shown in g (j) bar graphs with scatter plots summarizing areas of anti-TRPV4-positive immunoreactivity shown in h. §, P<0.05; §§§, P<0.005 as compared to the matching areas in i.

Table S1.1. Two factor characteristics of seven human genes (Lucas et al., 2021;McKnight et al., 2021;Raines et al., 2022)

Human gene	gene ID	chr*	gene locus	telomere locus	gene to telomere	A, T ** (%)	A + T (%)	FL*** (bp)
1	*TRPV4*	12	109K	133K	24	18,23	41	3228
2	*ZCCHC8*	12	122	133	11	30,28	58	4113
3	*VEGF*	6	43	0	43	24,26	50	3609
4	*TBXAS1*	7	140	159	19	22,26	48	1923
5	*MAPT*	17	45	82	37	22,23	45	5639
6	*APP*	21	26	46	20	27,25	52	3583
7	*GAPDH*	12	6	0	6	23,22	45	1285

Table S1.2. Two factor characteristics of seven rat genes

Rat gene	gene ID	chr*	gene locus	telomere locus	gene to telomere	A, T ** (%)	A + T (%)	FL*** (bp)
1	*TRPV4*	12	41	46	5	20,23	43	3211
2	*ZCCHC8*	12	32	46	14	27,25	52	2818
3	*VEGF*	9	14	114	100	25,26	51	3546
4	*TBXAS1*	4	67	182	115	23,27	50	1849
5	*MAPT*	10	89	107	18	24,24	48	5149
6	*APP*	11	24	86	62	26,21	47	2340
7	*GAPDH*	4	157	182	25	25,22	47	1306

Table S1.3. Two factor characteristics of seven mouse genes

Mouse gene	gene ID	chr*	gene locus	telomere locus	gene to telomere	A, T ** (%)	A + T (%)	FL*** (bp)
1	*TRPV4*	5	114	151	37	20,22	42	3247
2	*ZCCHC8*	5	123	151	28	26,27	53	4286
3	*VEGF*	17	46	95	49	25,25	50	3475
4	*TBXAS1*	6	38	149	111	24,25	49	1992
5	*MAPT*	11	104	122	18	24,25	49	5164
6	*APP*	16	84	97	18	27,23	50	3152
7	*GAPDH*	6	125	149	24	24,22	46	1444

Table S2 Inclusion criteria: human postmortem tissues from the NIH NBB

Criteria	Tissue	RNA integrity number (RIN)	Age	Type	Sex	HIV*	HBSAG**	PMInterval***
Include	Cortex, caudate nucleus, cerebellum	6 - 7.2	≥ 65 yr	Frozen	Male & female	Negative	Negative	< 36 hr
Exclude	elsewhere	<6 or >7.2	< 65 yr	Fixed	-	Positive	Positive	≥ 36 hr

* Human immunodeficiency virus; ** Hepatitis B Surface Antigen Test; *** Postmortem interval

Table S3 Postmortem specimen information

Numbering	Subject ID	Age (years)	Disorder	Sex	Race	Medical History
1	5219	76	Unaffected Control	Female	White	
2	4921	73	Unaffected Control	Female	White	* gall bladder problem
3	3642	88	Unaffected Control	Female	White	** cong. heart failure
4	4789	72	Unaffected Control	Female	White	
5	5671	78	Unaffected Control	Male	White	
6	s06424	71	Unaffected Control	Female	White	
7	42055	85	Unaffected Control	Male	White	
8	s08544	72	NPH	Female	White	[a]
9	994765	86	NPH	Male	White	[b]
10	80455	85	NPH	Male	White	[c]
11	4922	79	NPH	Male	White	[d] TBI
12	3924	79	NPH	Female	White	[e] MS
13	3637	76	NPH	Male	White	[f] cerebral atherosclerosis.
14	21762	77	NPH	Female	African American	[g] vascular dementia
15	1212	72	AD	Female	White	
16	5584	88	AD	Female	White	
17	5501	78	AD	Female	White	
18	6560	67	AD	Female	White	
19	5914	83	AD	Male	White	

* Donor also with high blood pressure. She collapsed while in the hospital. She was still breathing when found on the floor but with no spontaneous respiration and no cardiac pulse. No further medical history available. "

** The decedent was an 88-year-old Caucasian female with a cardiac hx whose cause of death was attributable to congestive heart failure. Neuropathology Diagnosis: Normal brain."

[a-c] idiopathic.

[d] personal history of traumatic brain injury (TBI), cerebral atherosclerosis, cerebral infarction (unspecified)

[e] Multiple sclerosis (MS), diagnostic pathology not present

[f] cerebral atherosclerosis, diagnostic pathology not present

[g] vascular dementia without behavioral disturbance

Table S4 Primer sequence for *human* gene transcripts

**MAPT** (product size: 232 b); Exon 6<br>(upstream) CTCCAAAATCAGGGGATCG<br>(downstream) TTCTCAGTGGAGCCGATCTT	**APP** (product size: 241 b); Exon 17<br>(upstream) TTAAGAATCGATGGGGGATG<br>(downstream) GCACATAAAACAGGCACGAA
**TBXAS1** (product size: 162 b); Exon 13<br>(upstream) CTCCTCTACTGGGTGCAAGC<br>(downstream) GCCAGTAGGTAGGTGGCAAA	**VEGF** (product size: 165 b); Exon 1<br>(upstream) CCGGGTTTTATCCCTCTTC<br>(downstream) TCTGCTGGTTTCCAAAATCC
**ZCCHC8** (product size: 191 b); Exon 1<br>(upstream) GCATTCCGGGAGTTGTAGTC<br>(downstream) TTGGGCTGTGGAAAAGATTC	**TRPV4** (nucleotide product size: 204 bases, b); Exon 2<br>(upstream) ATCTGTTTGAGGGGGAGGAT<br>(downstream) AGTCCATGGGTGCTTTCTTG
**GAPDH** (product size: 201 b); Exon 6<br>(upstream) ACCCAGAAGACTGTGGATGG<br>(downstream) TTCTAGACGGCAGGTCAGGT	

References for supplementary material

Hastbacka, J., De La Chapelle, A., Kaitila, I., Sistonen, P., Weaver, A., and Lander, E. (1992). Linkage disequilibrium mapping in isolated founder populations: diastrophic dysplasia in Finland. *Nat Genet* 2**,** 204-211.

Lucas, H.B., Mcknight, I., Raines, R., Hijazi, A., Hart, C., Lee, C., Kim, D.G., Li, W., Lee, P.H.U., and Shim, J.W. (2021). Factors Associated with Mutations: Their Matching Rates to Cardiovascular and Neurological Diseases. *Int J Mol Sci* 22.

Mcknight, I., Hart, C., Park, I.H., and Shim, J.W. (2021). Genes causing congenital hydrocephalus: Their chromosomal characteristics of telomere proximity and DNA compositions. *Exp Neurol* 335**,** 113523.

Nusbaum, C., Mikkelsen, T.S., Zody, M.C., Asakawa, S., Taudien, S., Garber, M., Kodira, C.D., Schueler, M.G., Shimizu, A., Whittaker, C.A., Chang, J.L., Cuomo, C.A., Dewar, K., Fitzgerald, M.G., Yang, X., Allen, N.R., Anderson, S., Asakawa, T., Blechschmidt, K., Bloom, T., Borowsky, M.L., Butler, J., Cook, A., Corum, B., Dearellano, K., Decaprio, D., Dooley, K.T., Dorris, L., 3rd, Engels, R., Glockner, G., Hafez, N., Hagopian, D.S., Hall, J.L., Ishikawa, S.K., Jaffe, D.B., Kamat, A., Kudoh, J., Lehmann, R., Lokitsang, T., Macdonald, P., Major, J.E., Matthews, C.D., Mauceli, E., Menzel, U., Mihalev, A.H., Minoshima, S., Murayama, Y., Naylor, J.W., Nicol, R., Nguyen, C., O'leary, S.B., O'neill, K., Parker, S.C., Polley, A., Raymond, C.K., Reichwald, K., Rodriguez, J., Sasaki, T., Schilhabel, M., Siddiqui, R., Smith, C.L., Sneddon, T.P., Talamas, J.A., Tenzin, P., Topham, K., Venkataraman, V., Wen, G., Yamazaki, S., Young, S.K., Zeng, Q., Zimmer, A.R., Rosenthal, A., Birren, B.W., Platzer, M., Shimizu, N., and Lander, E.S. (2006). DNA sequence and analysis of human chromosome 8. *Nature* 439**,** 331-335.

Raines, R., Mcknight, I., White, H., Legg, K., Lee, C., Li, W., Lee, P.H.U., and Shim, J.W. (2022). Drug-Targeted Genomes: Mutability of Ion Channels and GPCRs. *Biomedicines* 10.
